# Whole Exome Sequencing of Extreme Morbid Obesity Patients: Translational Implications for Obesity and Related Disorders

**DOI:** 10.3390/genes5030709

**Published:** 2014-08-25

**Authors:** Gilberto Paz-Filho, Margaret C.S. Boguszewski, Claudio A. Mastronardi, Hardip R. Patel, Angad S. Johar, Aaron Chuah, Gavin A. Huttley, Cesar L. Boguszewski, Ma-Li Wong, Mauricio Arcos-Burgos, Julio Licinio

**Affiliations:** 1 Genome Biology Department, The John Curtin School of Medical Research, The Australian National University, Garran Rd, building 131, Acton, Canberra, ACT 0200, Australia; E-Mails: gilberto.pazfilho@anu.edu.au (G.P.-F.); Claudio.mastronardi@anu.edu.au (C.A.M.); Hardip.Patel@anu.edu.au (H.R.P); u4842377@anu.edu.au (A.S.J.); Aaron.Chuah@anu.edu.au (A.C.); gavin.huttley@anu.edu.au (G.A.H.); Mauricio.Arcos-Burgos@anu.edu.au (M.A.-B.); 2 Endocrine Division (SEMPR), Department of Internal Medicine, Federal University of Parana, Avenida Agostinho Leão Junior, 285-Alto da Glória. CEP 80030-110, Curitiba-PR, Brazil; E-Mails: margabogus@uol.com.br (M.C.S.B.); clbogus@uol.com.br (C.L.B.); 3 Mind and Brain Theme, South Australian Health and Medical Research Institute, and Department of Psychiatry, School of Medicine, Flinders University, PO Box 11060 Adelaide SA 5001, Adelaide, Australia; E-Mail: maliwong7@gmail.com (M.-L.W.)

**Keywords:** DNAAF1, genetics, leptin-melanocortin, LRP2, megalin, monogenic, obesity, pituitary, UCP2, whole-exome sequencing

## Abstract

Whole-exome sequencing (WES) is a new tool that allows the rapid, inexpensive and accurate exploration of Mendelian and complex diseases, such as obesity. To identify sequence variants associated with obesity, we performed WES of family trios of one male teenager and one female child with severe early-onset obesity. Additionally, the teenager patient had hypopituitarism and hyperprolactinaemia. A comprehensive bioinformatics analysis found *de novo* and compound heterozygote sequence variants with a damaging effect on genes previously associated with obesity in mice (*LRP2*) and humans (*UCP2*), among other intriguing mutations affecting ciliary function (*DNAAF1*). A gene ontology and pathway analysis of genes harbouring mutations resulted in the significant identification of overrepresented pathways related to ATP/ITP (adenosine/inosine triphosphate) metabolism and, in general, to the regulation of lipid metabolism. We discuss the clinical and physiological consequences of these mutations and the importance of these findings for either the clinical assessment or eventual treatment of morbid obesity.

## 1. Introduction 

Obesity is a global epidemic: the World Health Organization (WHO) estimates that half a billion people over the age of twenty worldwide are obese [1]. Global projections estimate that worldwide, 1.2 billion individuals will be obese by 2030 [2].

Obesity and its related traits have high estimates of heritability (h^2^ between 40% and 70%) [3]. The investigation of candidate genes and genome-wide association studies have identified more than 60 obesity susceptibility genes that predispose to increased body weight, waist circumference, waist-hip ratio, body mass index (BMI) and fat percentage or fat mass. However, mutations in these genes account for a very small fraction of the obesity phenotypic variance [4,5]. It has been estimated that at least 7% of children with severe early-onset obesity (defined by an onset before the age of 10 years and BMI over three standard deviations (SD) above normal) have a single locus sequence variant determining obesity [6]. Nine susceptibility genes, determinants of non-syndromic Mendelian forms of human obesity, are involved in the hypothalamic control of energy balance via the leptin-melanocortin pathway and/or in neural development [4]: brain-derived neurotrophic factor (BDNF), leptin (LEP), leptin receptor (LEPR), melanocortin-4 receptor (MC4R), neurotrophic tyrosine kinase receptor type 2 (NTRK2), prohormone convertase 1 (PCSK1), proopiomelanocortin (POMC), single-minded homolog 1 (SIM1) and, more recently, melanocortin 2 receptor accessory protein 2 (MRAP2) [7].

Exome sequencing is rapidly becoming the first-line approach for monogenic disorders [8]. The use of whole-exome capture and the complete sequencing of the coding genome of parent-child trios is a highly effective approach for identifying homozygous, compound heterozygous and *de novo* coding sequence variants, as multiple *de novo* sequence variants occurring within a specific gene (or within a gene family or pathway) are extremely implausible events [8]. Its rationale is based on the fact that gene variants located in exons are more likely to be pathogenic than those located in introns or between genes. The power of this strategy has increased with the access to large numbers of publicly available exome sequences that allow the controlled comparison of frequencies, as well as the identification of *de novo* variants and stratification by ethnicity. This strategy has been used to identify candidate genes for several Mendelian and complex traits [9,10,11,12]. In the assessment of obesity, whole-exome sequencing has identified sequence variants in the leptin receptor gene [13], in the *ADCY3* gene [14] and in the *BBIP1* gene in patients with Bardet–Biedl syndrome [15]. However, no novel pathogenic genes or pathways associated with obesity have been identified through this approach yet. 

In this study, by employing whole-exome capture and sequencing in the assessment of two patients with severe early-onset obesity (and their parents), we identified *de novo* mutations and the compound heterozygous status of several damaging variants. Intriguingly, some of these variants were harboured in genes involved in the pathophysiology of obesity (such as *LRP2* and *UCP2*), providing the foundation for future research in this field. Thus far, we emphasize that the networking of clinical case-reports and genetic analyses would be crucial to finding the major loci underpinning complex disorders. 

## 2. Experimental

Two family trios, the probands of which had severe early-onset obesity (onset before the age of 10 years and BMI over three SD above normal) were included in this study. All parents and capable patients provided written informed consent for the genetic research studies, which were performed in accordance with the study protocol approved by the Australian National University Human Research Ethics Committee (Protocol 2011/108, approved on the 6 May, 2011) and in concordance with the Helsinki Declaration of 1975, as revised in 2008. DNA was extracted from peripheral blood from the patients and parents for genetic analysis.

### 2.1. DNA Library Preparation, Exome Capture and Sequencing Protocol

Libraries were constructed from 1 μg of genomic DNA using an Illumina TruSeq genomic DNA library kit (Illumina Inc., San Diego, CA, USA). Libraries were multiplexed with 6 samples pooled together (500 ng of each library). Exons were enriched from the pooled 3 μg of library DNA using an Illumina TruSeq Exome enrichment kit (Illumina Inc.). Each exome-enriched pool was run on a 100-base-pair paired-end run on an Illumina HiSeq 2000 sequencer (Illumina Inc.). We surveyed 201,071 genomic regions in total using the exome capture platform. Ninety percent of the bases in approximately 197,000 of these targeted regions had at least one read coverage. All regions were sampled at approximately 50× coverage.

### 2.2. Sequence Read Processing, Alignment, Bioinformatics and Genetic Analyses

The sequencing image data were processed in real time using Illumina Real Time Analysis (RTA) software (Illumina Inc.) and converted to fastq files containing DNA base calls (A, C, G and T) and quality scores using the Illumina CASAVA pipeline (a software program that converts raw image data into sequences). The resulting fastq files were further processed for variant analysis.

The entire workflow of data curation and analysis for variant-calling was developed by the Genome Discovery Unit (GDU) at The Australian National University. Key components of the workflow include: (i) quality assessment; (ii) read alignment; (iii) local realignment around the known and novel indel regions to refine indel boundaries; (iv) recalibration of base qualities; (v) variant calling; and (vi) assigning quality scores to variants (detailed workflow information is in the Supplemental Material). 

Subsequently, we included a filtering phase (using information from dbSNP and the 1K Exome Project), with the following sequential steps: (1) identification of rare or *de novo* variants (a lower minor allele frequency cut-off (MAF) in the window of 0.1%–1.0%); (2) filtering of variants to include those that are potentially pathogenic or are specific variants associated with disease susceptibility using several tools, namely, SIFT, PolyPhen2, Mutation Taster, Mutation Assessor and Functional Analysis through Hidden Markov Models (FATHMM), as implemented by the DNA-seq Analysis Package (SVS7.7.6, Golden Helix, Bozeman, MT, USA) (variants were not excluded if classified as potentially damaging by at least one of these filtering tools); (3) filtering of damaging variants based on genes known to be associated with human disease; and (4) independent confirmation of selected variants by Sanger sequencing (Supplemental Material). The definition of *de novo* sequence variants, compound heterozygous polymorphisms and rare recessive homozygous polymorphisms was performed with different modules of the DNA-seq Analysis Package (SVS7.7.6, Golden Helix, Bozeman, MT, USA).

To identify potential enriched endocrine-physiological pathways, a genetic ontology pathway analysis was performed. For constructing the pathways, variants with potential functional changes detected by the *de novo* and the compound heterozygous analysis were examined with the set of algorithms implemented in MetaCore (Thomson Reuters, New York, NY, USA) for the heuristic interpretation of maps, networks and rich ontologies for diseases.

## 3. Clinical Reports

**Figure 1:**
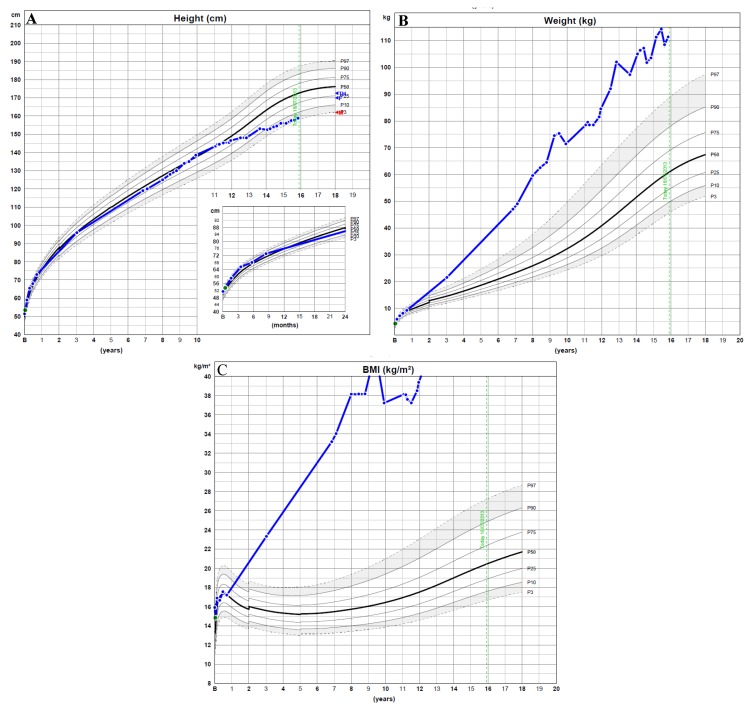
Height, weight and BMI of Patient 1, from birth to age 15 y, 10 m.

Patient 1: A Brazilian male teenager with a history of excessive weight gain starting at age 3, decelerated growth since age 11 and delayed puberty was first evaluated at age 14 y, 3 m. His body weight was 105.0 kg (+5.32 SD score), his height 152.5 cm (−1.45 SD score) and his BMI 45.2 kg/m^2^ (+11.03 SD score) (Figure 1). The patient had no complaints of hearing deficits or vision loss. Testicular volumes were <2 mL bilaterally, and he was at Tanner pubertal stage P2–P3. He had cubitus valgus and round facies. During physical examination, profuse sweating was noted. Other physical signs were unremarkable.

Growth charts illustrating changes in height (**A**), weight (**B**) and BMI (**C**). M, maternal height; P, paternal height; TH, target height. From WHO Child Growth Standard: Methods and Developments.

Obesity was a common finding in his family, but all individuals had normal height. His father is obese (BMI 36.7 kg/m^2^), as are his paternal grandfather (BMI 44.6 kg/m^2^) and paternal uncle (BMI 50.5 kg/m^2^). His mother’s BMI is 30.1 kg/m^2^, and two maternal aunts are also obese (BMI 31.6 and 30.1 kg/m^2^). His older brother is overweight (BMI 29 kg/m^2^). None of his family members have a history of severe early-onset obesity. There was no history of consanguinity in the family. Pregnancy was uneventful, and size at birth was 4.2 kg and 51.5 cm. 

He had been previously diagnosed with central hypothyroidism at age 9, with thyroid-stimulating hormone (TSH) of 7.4 mU/L and free T4 of 9.5 pmol/L, with undetectable titres of antithyroglobulin and antithyroperoxidase antibodies and a normal thyroid ultrasound. He also had elevated serum prolactin levels of 2,908 pmol/L, measured for the first time at age 9. Magnetic resonance enhanced by the contrast gadolinium showed a pituitary gland of normal volume, with no evidence of pituitary adenoma and without any structural abnormalities in the brain. The search for macroprolactin was negative (71% recovery after polyethylene glycol precipitation). He had been on treatment with levothyroxine 88 μg/day since age 9 y, 5 m, and cabergoline 0.5 mg every 10–15 days since age 13 y, 2 m, which had normalized his serum TSH and prolactin levels.

During the previous five years, he was treated with hypocaloric mixed diets, frequent physical activity, sibutramine 10 mg/day and orlistat 120 mg after meals. This approach resulted in only a 6-kg noncontinuous weight loss. 

The patient’s serum triglycerides and total cholesterol were elevated (3.11 and 4.84 mmol/L, respectively), with low HDL-cholesterol of 0.85 mmol/L and normal calculated LDL-cholesterol of 1.99 mmol/L. Fasting plasma glucose and insulin were 5.33 mmol/L and 13 μU/mL, respectively, with the homeostasis model assessment-estimated insulin resistance (HOMA-IR) index equal to 3.07. His serum insulin-like growth factor 1 (IGF-1) level was below the age reference range (71.25 nmol/L; reference value 130–563 nmol/L). Growth hormone (GH) secretion was evaluated during a standard insulin provocative test, with no development of hypoglycaemia during the test (lowest glucose level of 3.55 mmol/L and respective GH level of 0.06 μg/L). Morning cortisol and adrenocorticotropic hormone (ACTH) levels were normal (0.68 μmol/L and 1.60 pmol/L, respectively). His serum leptin levels were 8.1 and 18.0 μg/L at age 9 and appropriately elevated at age 13 (69.7 μg/L). At age 13, his total testosterone levels were pre-pubertal (0.90 nmol/L), and follicle-stimulating hormone (FSH) and luteinizing hormone (LH) were undetectable. At age 13 y, 9 m, his bone age was 15.6 years. Table 1 summarizes the laboratory test results and their respective reference range values. 

**Table 1:** Laboratory test results for Patient 1.

Test	Value	Normal reference range
Thyroid-stimulating hormone (TSH) *	7.4 mU/L	0.3–5.0 mU/L
Free T4 *	9.5 pmol/L	10.3–25.7 pmol/L
Antithyroglobulin (ATG) and antithyroperoxidase (ATPO) antibodies *	Both negative	<9.0 IU/mL (ATG)<116 IU/mL (ATPO)
Prolactin *	2908 pmol/L	82–504 pmol/L
Macroprolactin *	Negative (71% recovery)	>50% recovery
Total cholesterol ^$^	4.84 mmol/L	4.4 mmol/L
HDL cholesterol ^$^	0.85 mmol/L	>1.16 mmol/L
LDL cholesterol ^$^	1.99 mmol/L	<2.84 mmol/L
Triglycerides ^$^	3.11 mmol/L	<1.02 mmol/L
Fasting plasma glucose ^$^	5.33 mmol/L	3.89–5.5 mmol/L
Fasting insulin ^$^	13 μU/mL	1.8–4.6 μU/mL
Insulin-like growth factor 1 (IGF-1) ^&^	71.25 nmol/L	130–563 nmol/L
Growth hormone (GH)/glucose ^&#^	0.06 μg/L/3.55 mmol/L	>5 μg/L/<1.94 mmol/L
Adrenocorticotropic hormone (ACTH) (morning)	1.60 pmol/L	2.2–13.2 pmol/L
Cortisol (morning)	0.68 μmol/L	0.14–0.70 μmol/L
Leptin	8.1 * and 69.7 μg/L ^&^	Detectable
Total testosterone ^&^	0.90 nmol/L	3.47–41.60 nmol/L
Follicle-stimulating hormone (FSH) ^&^	Undetectable	0.5–10.5 IU/L
Luteinizing hormone (LH) ^&^	Undetectable	0.5–7.9 IU/L
Total calcium ^	2.62 mmol/L	2.40–2.64 mmol/L
Inorganic phosphate ^	173.4 mmol/L	108.4–164.2 mmol/L
Magnesium ^	1.1 mmol/L	0.7–0.9 mmol/L
Alkaline phosphatise ^	114 U/L	66–571 U/L
25-hydroxy vitamin D ^	85 mmol/L	>75 mmol/L
Parathyroid hormone (PTH) ^	2.6 pmol/L	1.0–5.5 pmol/L
Selenium ^@^	0.03 μmol/L	0.25–2.4 μmol/L
Total urinary protein ^@^	0.08 g/24 hours	<0.15 g/24 hours

* Measured at age 9, not treated with levothyroxine and cabergoline; ^$^ measured at age 12; ^#^ GH and lowest glucose level measured during a standard insulin provocative test; ^&^ measured at age 13; ^ measured at age 14; ^@^ measured at age 16.

Radiographic studies showed lumbar scoliosis convex to the right, mild reduction of intervertebral spaces at L4-S1, mild shortening of L1, as well as a short fourth metacarpal. In order to exclude the diagnosis of Albright’s hereditary osteodystrophy, serum electrolytes, alkaline phosphatase, vitamin D and parathyroid hormone (PTH) were measured, which were all unremarkable for Albright’s hereditary osteodystrophy (total calcium 2.62 mmol/L; inorganic phosphate 173.4 mmol/L; magnesium 1.1 mmol/L, alkaline phosphatase 114 U/L, 25-hydroxy vitamin D 85 mmol/L and PTH 2.6 pmol/L). His serum levels of selenium were 0.03 μmol/L, 10-fold lower than the lowest limit of the normal range (reference values 0.25–2.4 μmol/L). Total urinary protein levels were 0.08 g/24 hours. 

Intramuscular injections of testosterone esters (70 mg every four weeks) were initiated for puberty induction. Biosynthetic GH was initiated in a dose of 0.33 mg/day and increased to 0.66 mg/day according to IGF-1 levels. Four months after starting GH therapy, he developed episodes of fever of unexplained origin, which resolved spontaneously after six months. During that period, investigation for infection disease was negative, including a normal PET-scan, with leucocytosis as the only observed abnormality. 

Patient 2: A two year-old Brazilian girl was evaluated for severe early-onset obesity. Her body weight was 23 kg (+4.79 SD score), her height was 93 cm (+2.32 SD score) and her BMI was 26.6 kg/m^2^ (+4.49 SD score). She was born with 2.9 kg and 46 cm, from an uneventful pregnancy. Excessive weight gain was noted upon a few weeks after birth. Neurologic development was normal, with no evidence of Prader–Willi or Bardet–Biedl syndromes. She had normal serum leptin levels of 18 μg/L. A history of recurrent bacterial and viral respiratory tract infections was noted, which warranted the need for antibiotic therapy almost every month. There was no significant familial history of obesity or consanguinity. The physical examination was unremarkable.

## 4. Results

We called a total of 455,342 variants, 336,652 of them polymorphic, 21,613 matched at the dbNSFP, 12,286 with potential pathogenic effects and 2,291 with a minor allele frequency <1% when compared to the 1 kG phase 1. Our filtering approach by *de novo* functional mutation screening reported three *de novo* sequence variants with a potential damaging effect (Table 2). These sequence variants were found in Patient 1 (*UCP2*) and in Patient 2 (*AICDA* and *FAM71E2*).

The compound heterozygous analysis identified 20 variants with potential functional effects associated with eight genes, namely: *LRP2*,* AMPD3*,* OR8U8-OR9G1* and* SLC22A6* in Patient 1 and* TTN*,* APEH*, *DNAAF1* and* KIR3DL3* in Patient 2 (Table 3). From a literature search on the compound heterozygous variants that were classified as damaging, we identified two sequence variants in the *LRP2* gene that may potentially affect LRP2 protein function in Patient 1: a genomic variant G→T (NC_000002.11:g.170009391G>T), resulting in a nonsynonymous substitution on codon 4127 (NM_004525.2:c.12379C>A; NP_004516.2:p.Arg4127Ser), and a genomic variant C→T (NC_000002.11:g.170030506C>T), resulting in a nonsynonymous substitution on codon 3646 (NM_004525.2:c.10937G>A; (NP_004516.2:p.Arg3646His). The *LRP2* gene encodes a multi-ligand endocytic receptor (also known as megalin or glycoprotein 330) involved in the regulation of the leptin-melanocortin pathway. 

**Table 2:** *De novo* damaging sequence variants.

Patient	Variant	Chr	Position	Ref All	Alt All	Identifier	Classification	Gene	Transcript	Exon	HGVS Coding	HGVS Protein
1	11:73689104-SNV	11	73,689,104	G	A	rs660339	Nonsyn SNV	UCP2	NM_003355	4	c.164C>T	p.Ala55Val
2	12:8757523-Ins	12	8,757,523	-	A	rs5796316	Splicing	AICDA	NM_020661	4	c.428-5_428-4insT	
2	19:55873642-SNV	19	55,873,642	C	T	rs4252574	Nonsyn SNV	FAM71E2	NM_001145402	3	c.535G>A	p.Glu179Lys

Chr: chromosome; Ref All: reference allele; Alt All: alternate allele; HGVS: Human Genome Variation Society nomenclature.

**Table 3:** Sequence rare variants (allele frequency in the general population <1%) with potential damaging effect by compound heterozygous analysis.

Patient	Single Nucleotide Variant	Chromosome	Position	Identifier	Gene	dbSNP MAF Frequency	Alleles	ReferenceAllele	Reference Aminoacid	Altered Aminoacid	HGVS Protein
1	2:170009391-SNV	2	170009391	rs148356370	LRP2	0.005	G/T	G	R	S	p.R4127S
1	2:170030506-SNV	2	170030506	rs142549310	LRP2	0.002	C/T	C	R	H	p.R3646H
1	11:10518373-SNV	11	10518373	rs144107914	AMPD3	0.001	C/T	C	S	L	p.S323L
1	11:10527316-SNV	11	10527316	N/A	AMPD3	***	A/G	G	R	Q	p.R571Q
1	11:56468198-SNV	11	56468198	rs4990194	OR8U8-OR9G1	0.069	A/G	A	Y	C	p.Y112C
1	11:56468212-SNV	11	56468212	rs591369	OR8U8-OR9G1	**	A/G	G	V	M	p.V117M
1	11:56468554-SNV	11	56468554	rs12420076	OR8U8-OR9G1	0.061	A/C	A	K	Q	p.K231Q
1	11:56468560-SNV	11	56468560	rs10896516	OR8U8-OR9G1	**	C/T	T	Y	H	p.Y233H
1	11:56468561-SNV	11	56468561	rs10896517	OR8U8-OR9G1	0.047	A/G	A	Y	C	p.Y233C
1	11:62748503-SNV	11	62748503	rs150409056	SLC22A6	*	G/T	G	R	S	p.R331S
1	11:62749384-SNV	11	62749384	rs200609617	SLC22A6	***	C/T	C	A	T	p.A243T
2	2:179507021-SNV	2	179507021	N/A	TTN	***	G/A	G	R	C	p.R4436C
2	2:179577628-SNV	2	179577628	N/A	TTN	***	C/T	C	V	I	p.V7798I
2	2:179634421-SNV	2	179634421	rs200875815	TTN	***	T/G	T	T	P	c.8749A>C
2	3:49716372-SNV	3	49716372	N/A	APEH	***	A/G	G	R	H	p.R383H
2	3:49720698-SNV	3	49720698	N/A	APEH	***	A/G	G	A	T	p.A708T
2	16:84203467-SNV	16	84203467	rs143322223	DNAAF1	***	C/G	C	E	Q	p.E345Q
2	16:84208329-SNV	16	84208329	rs139519641	DNAAF1	*	A/G	A	?	?	Splicing
2	19:55239223-SNV	19	55239223	rs117372288	KIR3DL3	***	A/G	A	V	I	p.V168I
2	19:55241240-SNV	19	55241240	rs111516669	KIR3DL3	**	A/G	A	V	I	p.V313I

Note: The first two rows show the sequence variants that affect function, harboured at the *LRP2* gene (NC_000002.11:g.170009391G>T and NC_000002.11:g.170030506C>T). N/A: not available. * Monomorphic in available data from dbSNP database; ** MAF only available for 2 chromosomes; *** There is no frequency data.

**Figure 2:**
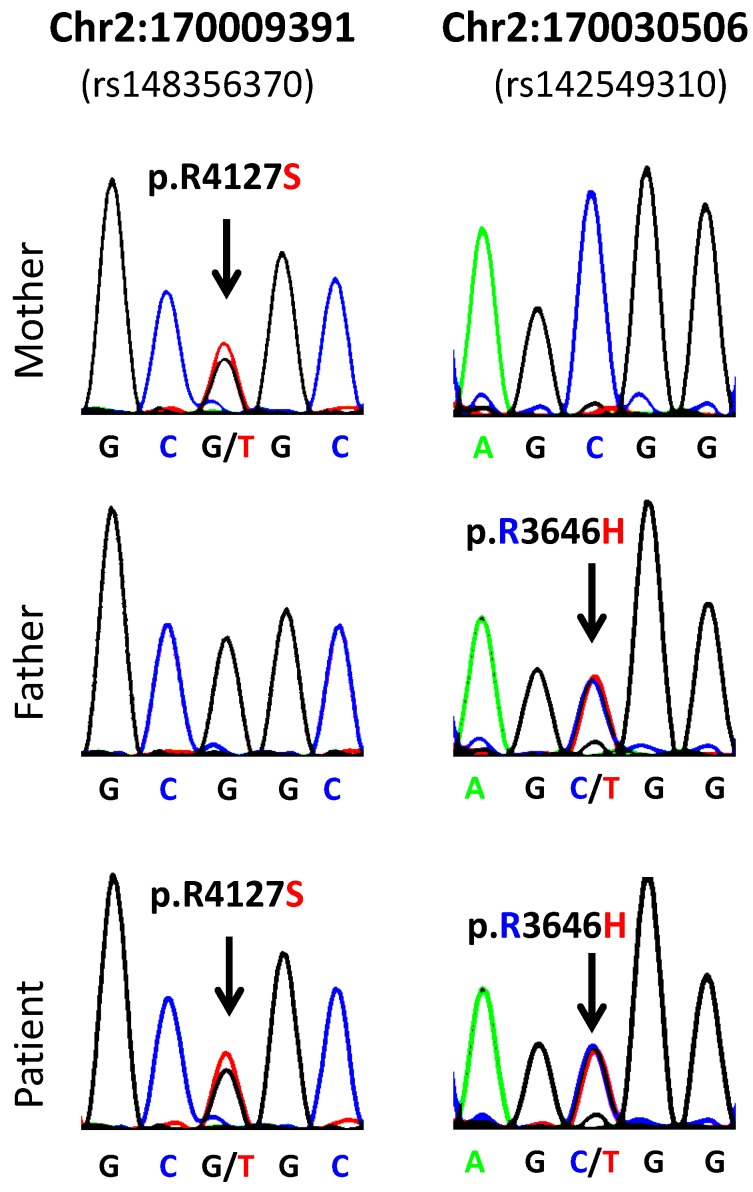
Sanger sequencing results from Patient 1 and parents.

Sanger sequencing of the two amplicons containing the two *LRP2* sequence variants validated the findings obtained from the whole-exome capture and sequencing analysis (Figure 2). These results confirmed that each parent is heterozygous for one of the sequence variants (the mother is heterozygous for the mentioned sequence variant (g/t) (NC_000002.11:g.170009391G>T), and the father is heterozygous for the sequence variant (c/t) (NC_000002.11:g.170030506C>T), whereas the patient is heterozygous for both *LRP2* sequence variants. Therefore, the patient is compound heterozygous for the *LRP2* gene as initially described in the whole-exome capture and sequencing analysis (Figure 2).

Sanger sequencing of the *LRP2* gene shows that both parents are heterozygous for one of the mutations. The mother is heterozygous for the Chr2:170009391 sequence variant (g/t) (NC_000002.11:g.170009391G>T), and the father is heterozygous for the Chr2: 170030506 variant (c/t) (NC_000002.11:g.170030506C>T). The patient is compound heterozygous and has both sequence variants.

MetaCore analysis, including those genes harbouring functional mutations, namely *UCP2*,* AICDA*,* FAM71E2*,* TTN*, *APEH*,* DNAAF1*, *KIR3DL3*,* LRP2*,* AMPD3*,* OR8U8-OR9G1* and* SLC22A6*, defined six pathways significantly overrepresented (after false discovery correction, FDR), e.g.,: (1) ATP/ITP (adenosine/inosine triphosphate) metabolism; (2) regulation of lipid metabolism/peroxisome proliferator-activated receptor (PPAR) regulation of lipid metabolism; (3) development of insulin, IGF-1 and TNF-alpha in brown adipocyte differentiation; (4) mitochondrial dysfunction in neurodegenerative diseases; (5) oxidative stress role of Sirtuin1 and PGC1 alpha in the activation of the defence system; and (6) CTP/UTP (cytidine/uridine triphosphate) metabolism (Table 4).

**Table 4:** Significant pathways from MetaCore analysis of candidate morbid obesity genes.

Pathways of Candidate Morbid Obesity Genes	Genes from Input List in Pathway	*p*-Value	FDR
ATP, ITP metabolism	AMPD3	1.107e-3	6.640e-3
Regulation of lipid metabolism PPAR regulation of lipid metabolism	UCP2	1.851e-2	3.164e-2
Development of insulin, IGF-1 and TNF-alpha in brown adipocyte differentiation	UCP2	2.332e-2	3.164e-2
Mitochondrial dysfunction in neurodegenerative diseases	UCP2	2.594e-2	3.164e-2
Oxidative stress role of Sirtuin1 and PGC1 alpha in the activation of the defence system	UCP2	2.637e-2	3.164e-2
CTP UTP metabolism	AICDA	4.709e-2	4.709e-2

Note: *p*-values and false discovery (FDR) correction adjusting for multiple comparisons, representing the probability that these pathways generated from our candidate gene list would appear by coincidental chance from feeding a random set of genes.

## 5. Conclusions

In this study, by employing whole-exome capture and sequencing, we identified novel sequence variants in the *LRP2* gene that might be associated with the phenotype of severe early-onset obesity in Patient 1. Similar to the other genes associated with monogenic forms of obesity, *LRP2* is also involved in the regulation of the leptin-melanocortin pathway [16]. In addition, in Patient 1, we found a *de novo* sequence variant in the *UCP2* gene, a transporter protein present in the mitochondrial inner membrane that is a key regulator of energy balance, the variants of which have already been associated with obesity [17]. In Patient 2, we identified sequence variants in the *DNAAF1* gene, which might be related to the second patient’s phenotype. The *DNAAF1* gene is required for the stability of the ciliary architecture, and it has been demonstrated that ciliary dysfunction is associated with the pathogenesis of obesity [18,19].

Recently, LRP2 has been nominated as a novel appetite regulator responsible for generating satiety signals in hypothalamic neurons [16]. It is a multiligand endocytic receptor, a member of the low density lipoprotein receptor gene family, which binds a large variety of ligands. Leptin is one of its ligands; LRP2 mediates its reuptake in renal tubules [20] and promotes leptin transport across the choroid plexus [21]. LRP2 also binds to the long-form leptin receptor (LepRb), forming a complex that is co-localized and subjected to endocytosis in the hypothalamic neurons. Subsequently, the endocytosis of this co-localized complex leads to the activation of signal transducer and activator of transcription 3 (STAT3) signalling in hypothalamic neurons, including proopiomelanocortin (POMC)- and LepRb-expressing neurons. As a consequence, food intake and body weight are decreased. In the absence of functional LRP2, STAT3 signalling is decreased. Therefore, hunger is stimulated and satiety decreased [16].

The binding of LRP2 and LepRb is enhanced by clusterin, a sulphated glycoprotein widely expressed in hypothalamic areas involved in the regulation of food intake and energy metabolism [22]. Chronic intracerebroventricular (icv) administration of clusterin causes the reduction of food intake, body weight and epididymal fat mass [16]. LRP2 is expressed in rodent hypothalamus, and its inhibition by small interfering RNA significantly blunts the effects of clusterin icv injections on food intake and Stat3 activation [16]. Therefore, LRP2 acts as a key mediator of the food intake-suppressing effects of clusterin, and its absence can cause obesity in rodents (as previously demonstrated) [16]. 

Sequence variants in the *LRP2* gene have been previously associated with Donnai–Barrow/facio-oculo-acoustico-renal (DB/FOAR) syndrome [23]. The phenotype of this syndrome includes agenesis of the corpus callosum, developmental delay, enlarged anterior fontanelle, high myopia, hypertelorism, proteinuria and sensorineural hearing loss, but not obesity. In a review by Pober *et al.*, sensorineural hearing loss, high myopia and proteinuria were present in 100% of DB/FOAR syndrome cases. None of those features were present in Patient 1; therefore, we ruled this diagnosis out. Serum selenium levels in patients with DB/FOAR syndrome have not been reported, but it has been shown that LRP2 mediates the reuptake of selenoproteins in the kidney and that LRP2-mutant mice have low selenium serum levels due to the increased urinary excretion of selenoproteins [24]. Since the patient’s serum levels of selenium were 10-fold lower than those of the reference range, this finding supports the impairment of the biological function of LRP2. 

Besides suffering from severe early-onset obesity, Patient 1 also had pituitary dysfunction characterized by GH deficiency, central hypothyroidism and hypogonadotropic hypogonadism, with concomitant hyperprolactinaemia. It is unclear whether sequence variants in the *LRP2* gene can directly affect pituitary development and function. LRP2 is essential for brain development [25], as knock-out mice exhibit holoprosencephaly [26] and *Lrp2-*mutant mice have abnormal cortical axon development [27]. Humans with DB/FOAR syndrome have structural brain abnormalities, mainly agenesis of the corpus callosum and, in one reported case, empty sella turcica [28]. These support the hypothesis that *LRP2* sequence variants might lead to abnormalities in pituitary development and hypopituitarism. 

In addition, Patient 1 had a *de novo* variant in the *UCP2* gene. As a transporter protein that is expressed in the mitochondrial inner membrane, UCP2 decreases mitochondrial ATP production by mediating H^+^ leak across the inner membrane [17]. Patient 1 has a G to A substitution at rs660339, resulting in an Ala55Val substitution, which has been associated with obesity in diverse settings [29,30]. Moreover, polymorphisms at rs660339 may also affect metabolic efficiency in terms of energy expenditure [31]. It is noteworthy to mention that this *de novo* variant has a reported minor allele frequency of about 0.5% and that it is a site that mutates recurrently. 

The other *de novo* sequence variants that we found in Patient 2 (AICDA, Activation-Induced Cytidine Deaminase; and FAM71E2, Family with Sequence Similarity 71, Member E2) are protein coding genes for a RNA-editing deaminase and for a protein of unknown function, respectively. The variant rs5796316 in AICDA has been reported in one previous study. Whereas AICDA is possibly not implicated in the pathophysiology of obesity, the function of FAM71E2 is unknown to date. However, rs4252574 in FAM71E2 is quite common, and the variant A allele is the major allele reported at about 70%. 

In Patient 2, we did not find gene variants that would strongly explain the phenotype, as we did for Patient 1. However, we identified sequence variants harboured in the *DNAAF1* that are possibly implicated in the patient’s history of recurrent infections and severe obesity. That gene is responsible for encoding a protein that is cilium-specific and is required for the stability of the ciliary architecture. *DNAFF1* is one of the 21 genes in which mutations are associated with primary ciliary dyskinesia (PCD) [32]. It is unlikely that Patient 2 has PCD, given the absence of its clinical manifestations (bronchiectasis, defects in body situs and, later in life, infertility). However, it is possible that identified *DNAAF1* sequence variants are causing a milder form of PCD with recurrent airway infections and severe obesity. Ciliary dysfunction has been associated with severe early-onset obesity, and currently, it is known that two obesity syndromes are caused by mutations in genes regulating ciliary function: Bardet–Biedl syndrome and Alström syndrome [19]. It has been demonstrated that ciliary dysfunction leads to the development of obesity in animal models, due to diverse alterations in central and peripheral pathways regulating energy metabolism [18,33,34,35,36,37,38]. For Patient 2, electron microscopy would be useful in the assessment of the effect of the DNAAF1 gene variant on ciliary structure. Furthermore, as we have not confirmed the DNAAF1 variant by Sanger sequencing, we cannot confirm that it is in *trans* in the patient (*i.e.*, each parent contributing one of the variants).

It is unlikely that the other gene variants with potential damaging effect (Table 3) also play a role in the pathogenesis of obesity, since they are not related to the regulation of the leptin-melanocortin pathway. Particularly, *TTN* is a large gene, the variants of which are frequently unrelated to disease; and *OR8U8-OR9G1,* the variants of whichmost likely represent artefacts, possibly due to alignment problems.

By performing MetaCore pathway analysis of the genes harbouring functional mutations, we observed that six pathways are significantly overrepresented, all of them involving energy or ATP/ITP/CTP/UCP metabolism. The importance of UCP2 on energy metabolism was further strengthened by the observation that four of these pathways were centred on the *UCP2* gene. 

In whole-genome association studies (GWAS), the *LRP2* and the *DNAAF1* genes were previously associated with increased BMI in a British population, without reaching a level of significance that is relevant for GWAS [39]. Curiously, a higher significance level for a single nucleotide polymorphism (SNP) within the *LRP2* gene (*p* = 8.68 × 10^−6^) was found in a GWAS of patients with anorexia nervosa [40]. Although variants at *UCP2* rs660339 have been associated with increased BMI in Europeans [41], this finding was not replicated in a recent meta-analysis in populations representing four ethnicities [42]. 

Whole-exome capture and sequencing can be used with family-based phenotype ascertainment strategies (nuclear and extended families) to exploit parent-child transmission and relative-relative sharing/not sharing patterns, as well as with arbitrary strategies of phenotype dichotomization to increase efficiency. In an extreme phenotype study design, individuals who are at both ends of a phenotype distribution are selected for sequencing. It is assumed that alleles contributing to the trait in individuals who are at both ends of the phenotype distribution are enriched, and sequencing even a modest sample size can potentially identify novel candidate alleles. The same consideration is also applicable to additional alternatives aimed to identify *de novo* variants that involve the sequencing of parent-offspring trios in which only the offspring is affected. The clinical use of whole-exome capture and sequencing is promising, as demonstrated in a recent study that evaluated 250 patients with undiagnosed diseases: the success rate in obtaining a genetic diagnosis was as high as 25% in that study [12]. 

Recently, guidelines for investigating and reporting the causality of sequence variants in human disease have been published, to avoid an acceleration of false-positive reports of causality [43]. In our study, we comply with those guidelines, but we acknowledge that our findings are not sufficient to implicate those gene variants as determinants of the obese phenotype. The significance of our results can be limited due to the fact that the number of probands is very small. However, a similar approach has already been validated and published by other studies, such as the Finding of Rare Disease Genes (FORGE) Canada Consortium [44]. In addition, WES has been applied for the diagnosis of several diseases, as observed in many case reports with very small sample sizes [45]. Furthermore, despite the fact that our results have not been replicated yet in other obese individuals, they are important to raise awareness of the *LRP2* gene as a possible candidate as a novel monogenic cause of obesity. In addition, our results lack confirmation through functional data, which should be pursued in future studies. 

Whole-exome capture and sequencing analysis is a time- and resource-intense endeavour. Currently, we employ software that allows rapid selection of any genetic variant according to variant type, novelty (via screening public and private databases) and predicted protein effect. However, linking these results to phenotypic manifestations in a particular person is currently performed by a mixture of manual analysis using a number of additional databases (e.g., Human Genome Mutation Database, Online Mendelian Inheritance in Man (OMIM), PubMed and UCSC, among others). We built on existing analytic tools in order to rapidly detect and annotate genomic variants associated with human disease. We are aware that analytical criteria for filtering need to be flexible and up-to-date; therefore, we undertook a systematic upgrade and iterative processes of database evaluation by considering each filter.

In conclusion, by employing a novel and unique strategy for whole-exome capture and sequencing analysis of two trios comprised of patients with severe early-onset obesity, we have identified sequence variants in the *LRP2* and in the *UCP2* genes that might explain the phenotype of a patient with severe early-onset obesity, central hypothyroidism, hypogonadotropic hypogonadism, growth hormone deficiency and idiopathic hyperprolactinaemia. In addition, we identified a sequence variant in the *DNAAF1* gene that might be implicated in the development of severe obesity associated with ciliary dysfunction. Whereas *de novo* variants in the *UCP2* gene have already been associated with obesity, the role of *LRP2* and *DNAAF1* sequence variants in human obesity needs to be further investigated by functional studies, and the frequency and distribution of those sequence variants need to be evaluated in a larger number of obese individuals.

## Supplementary Files

Supplementary File 1
