# Supplementary material for: Whole Exome Sequencing of Extreme Morbid Obesity Patients: Translational Implications for Obesity and Related Disorders
Source: Genes (Basel). 2014 Aug 25;5(3):709–25. doi: 10.3390/genes5030709 (PMC4198926; doi:10.3390/genes5030709)
Supplement: Supplementary File 1 [file genes-05-00709-s001.docx]

**Supplemental Material**

**Bioinformatics Workflow**

(i) Quality control: trimming of adapters and low-quality bases via FASTX-Toolkit [1];

(ii) Short-read alignment: reads were mapped to the latest genome assembly using a number of genome alignment heuristic algorithms, presently Burrows–Wheeler Aligner, allowing the confirmation of read alignments across different aligners and increasing confidence in the accuracy of variant calls;

(iii) Variant calling: single nucleotide polymorphisms (SNPs) were called using samtools pileup [2], and Indels using Dindel [3]. As the field of structural variant (SV) discovery is still undergoing rapid development, we called SVs using established tools such as BreakDancerMax [4] and Pindel [5] while comparing results with newer tools such as inGAP-sv [6] and CREST [7];

(iv) Validation of variants: this was performed against local copies of published variation databases (via EnsEMBL, which collates the Human Gene Mutation Database, the National Center for Biotechnology Information’s (NCBI) dbSNP, and Online Mendelian Inheritance in Man (OMIM).

(v) Quality assurance on data sampling: in addition to basic quality control, we employed KING [8] and EIGENSTRAT [9] principal component analyses in our pipeline to validate population homogeneity of samples.

(vi) Prediction of functional impact of genetic variants: this was performed using SIFT (Sorting Intolerant from Tolerant) [10], PolyPhen2 (Polymorphism Phenotyping v2) [11], Mutation Taster [12], Mutation Assessor [13], phyloP (phylogenetic P-values) [14] and GERP (Genetic Evolutionary Rate Profiling) [15]. All of these strategies enrich for functional sites at which observed variants are more likely to affect phenotype.

**Sanger Sequencing**

Sanger sequencing was performed using the patient’s and the parents’ DNA for the validation of the two sequence variants described in the *LRP2* gene (NM_004525.2:c.12379C>A; NP_004516.2:p.Arg4127Ser, and NM_004525.2:c.10937G>A; NP_004516.2:p.Arg3646His) by whole-exome sequencing. Briefly, a flanking region around each sequence variant site was amplified by PCR with the following primer pairs: (a) sense primer (5'atgctggggtaagtggaggtagtt-3') and antisense primer (5'-ggagggaggtagagatgtcaataa-3') for Chr2:170009391; and (b) sense primer (5'-gccagagtttgcagggagtg-3') and antisense primer
(5'-caggtcagggtgttcagttcttat-3') for Chr2:170030506. The primers were designed with the PrimerSelect software from the Lasergene genomics suite (DNASTAR, Inc, Madison, WI, USA), and were checked for uniqueness by employing the Basic Local Alignment Search Tool (BLAST) search engine. We used the following PCR conditions for the amplification of both amplicons: (1) initial activation step of 3 min at 94 °C, (2) 40 cycles as follows: 45 s of denaturing at 94 °C, 30 s of annealing at 56 °C, 60 s of extension at 72 °C, and (3) final extension step of 10 min at 72 °C. A 15-µL aliquot of the PCR product was analysed by electrophoresis in a 1.5% agarose gel to confirm the expected size of the two amplicons [457 and 630 bp for (NP_004516.2:p.Arg4127Ser) and (NP_004516.2: p.Arg3646His), respectively]. Then, 85µL of each PCR product was purified with the Qiaquick nucleotide removal kit (Qiagen, Valencia, CA, USA) following the manufacturer’s guidelines. Thereafter, the purified PCR products were spectrophotometrically quantified with a NanoDrop ND-1000 (Wilmington, DE, USA), and sent for Sanger sequencing to the Australian Cancer Research Foundation—Biomolecular Resource Facility (BRF) at the John Curtin School of Medical Research. Bidirectional sequencing of PCR amplicons were carried out by using Big DyeTM chemistry (Big Dye Terminator, Version 3.1; Applied Biosystems, Foster City, CA, USA) with the following internal (nested) primers to ensure specificity: (a) sense (5'-ttcagcaagcccaaccact-3') and antisense (5'-acttcacctgattagacccctgtt-3') for the amplicon containing the (NP_004516.2:p.Arg4127Ser) sequence variant, and b) sense
(5'-catcccatcagctgaaaaagaaag-3') and antisense (5'-cttccaagctgataaccaaatgtc-3') for the amplicon containing the (NP_004516.2: p.Arg3646His) sequence variant. The sequencing protocol was followed according to the BRF standard operative procedures.

**References**

1. Gordon, A. *FASTX-Toolkit*; Cold Spring Harbor Lab: Cold Spring Harbor, NY, USA, 2010.
2. Li, H.; Handsaker, B.; Wysoker, A.; Fennell, T.; Ruan, J.; Homer, N.; Marth, G.; Abecasis, G.; Durbin, R. The Sequence Alignment/Map format and SAMtools. *Bioinformatics* **2009**, *25*,
   2078–2079.
3. Albers, C.A.; Lunter, G.; MacArthur, D.G.; McVean, G.; Ouwehand, W.H.; Durbin, R. Dindel: Accurate indel calls from short-read data. *Genome Res.* **2011**, *21*, 961–973.
4. Chen, K.; Wallis, J.W.; McLellan, M.D.; Larson, D.E.; Kalicki, J.M.; Pohl, C.S.; McGrath, S.D.; Wendl, M.C.; Zhang, Q.; Locke, D.P.; *et al*. Break dancer: An algorithm for high-resolution mapping of genomic structural variation. *Nat. Methods* **2009**, *6*, 677–681.
5. Ye, K.; Schulz, M.H.; Long, Q.; Apweiler, R.; Ning, Z. Pindel: A pattern growth approach to detect break points of large deletions and medium sized insertions from paired-end short reads. *Bioinformatics* **2009**, *25*, 2865–2871.
6. Qi, J.; Zhao, F. InGAP-sv: A novel scheme to identify and visualize structural variation from paired end mapping data. *Nucleic Acids Res.* **2011**, *39*, W567–W575.
7. Wang, J.; Mullighan, C.G.; Easton, J.; Roberts, S.; Heatley, S.L.; Ma, J.; Rusch, M.C.; Chen, K.; Harris, C.C.; Ding, L.; *et al*. CREST maps somatic structural variation in cancer genomes with base-pair resolution. *Nat.* *Methods* **2011**, *8*, 652–654.
8. Manichaikul, A.; Mychaleckyj, J.C.; Rich, S.S.; Daly, K.; Sale, M.; Chen, W.M. Robust relationship inference in genome-wide association studies. *Bioinformatics* **2010**, *26*, 2867–2873.
9. Price, A.L.; Patterson, N.J.; Plenge, R.M.; Weinblatt, M.E.; Shadick, N.A.; Reich, D. Principal components analysis corrects for stratification in genome-wide association studies. *Nat Genet.* **2006**, *38*, 904–909.
10. Sorting intolerant from tolerant 2013. Available online: http://sift.bii.a-star.edu.sg/ (accessed on 23 July 2014).
11. Adzhubei, I.A.; Schmidt, S.; Peshkin, L.; Ramensky, V.E.; Gerasimova, A.; Bork, P.; Kondrashov, A.S.; Sunyaev, S.R. A method and server for predicting damaging missense mutations. *Nat. Methods* **2010**, *7*, 248–249.
12. Schwarz, J.M.; Rodelsperger, C.; Schuelke, M.; Seelow, D. MutationTaster evaluates
    disease-causing potential of sequence alterations. *Nat. Methods* **2010**, *7*, 575–576.
13. Reva, B.; Antipin, Y.; Sander, C. Predicting the functional impact of protein mutations: Application to cancer genomics. *Nucleic Acids Res.* **2011**, *39*, e118.
14. Siepel, A., Pollard, K.S., Haussler, D., Eds. New methods for detecting lineage-specific selection. In *Research in Computational Molecular Biology*; Springer Berlin Heidelberg: Berlin, Heidelberg, Germany, 2006; Volume 3909, pp. 190–205.
15. Cooper, G.M.; Stone, E.A.; Asimenos, G.; Program, N.C.S.; Green, E.D.; Batzoglou, S.; Sidow, A. Distribution and intensity of constraint in mammalian genomic sequence. *Genome Res.* **2005**, *15*, 901–913.

© 2014 by the authors; licensee MDPI, Basel, Switzerland. This article is an open access article distributed under the terms and conditions of the Creative Commons Attribution license (http://creativecommons.org/licenses/by/3.0/).
